# The Pathogenesis of Rheumatoid Arthritis Breakthroughs in Molecular Mechanisms 1 and 2

**DOI:** 10.3390/ijms241311060

**Published:** 2023-07-04

**Authors:** Yuki Nanke

**Affiliations:** 1 Institute of Rheumatology, Tokyo Women’s Medical University, Tokyo 162-8666, Japan; ynn@twmu.ac.jp; 2 Division of Rheumatology, First Department of Comprehensive Medicine, Jichi Medical University Saitama Medical Center, Saitama 330-0834, Japan

The pathogenesis of rheumatoid arthritis (RA) consists of the formation of synovial villi, inflammation, immune abnormalities, and bone–cartilage destruction. According to these pathogenic findings, conventional therapy was empirically performed using conventional synthetic disease-modifying antirheumatic drugs (csDMARD). However, since the 1990s, pathogenesis investigations have advanced to include the cloning of IL-6, clarifying the role of IL-17·Th17 in bone destruction; cloning to RANKL; anti-RANKL Ab in therapy for RA in Japan; and the introduction of “osteoimmunology”.

In addition, therapies using biological DMARDs have resulted in breakthroughs in pathogenesis investigations; the inhibition of the function of a specific molecule by an antibody has clarified its function in vivo, such as “knock-out in vivo in humans”. Recently, inflammatory cell states have been clarified in RA joint synovial tissues by integrating single-cell transcriptomics and mass cytometry. In this Special Issue, both original and review papers present important advances in the molecular investigations of RA pathogenesis.

The new Special Issue in the *International Journal of Molecular Sciences*, entitled “The pathogenesis of Rheumatoid Arthritis- Breakthroughs in Molecular Mechanisms”, includes a total of 14 contributions (6 original articles and 8 reviews) providing new information about the pathogenesis and molecular mechanisms of RA.

The progressive destruction of synovial-lined joints is a hallmark of RA. The fibroblast-like synoviocytes (FLSs) are the main effectors of the degradation. Kadiri M et al. [1] showed that 14-3-3 n, which belongs to a family of ubiquitously expressed intracellular scaffolding proteins, promotes invadosome formation by increasing Snail expression, a mechanism that involves the nuclear exclusion of the transcription repressor FOXO3. The authors identified 14-3-3 n as a novel regulator of invadosome formation and matrix degradation by RA synovial cells. The 14-3-3n-FOXO3-Snail axis promotes the aggressive extracellular-matrix-degrading phenotype of RA FLS.

Neutrophils and their extracellular traps play important roles in the pathogenesis of RA. Previous reports have revealed that neutrophil extracellular trap (NET)osis is the main source of citrullinated autoantigens in RA. Ohyama et al. [2] identified a specific increase in NETosis in peptide GPI-induced arthritis (pGIA) joints. The administration of anti-IL-6 receptor antibodies decreased neutrophilic infiltration, NETosis in the joints, and plasma citrullinated protein. Thus, IL-6 signaling may play an important role in the production of citrullinated proteins via effects on neutrophil chemotaxis and their extracellular traps in RA.

Pietschmann et al. [3] reviewed the molecular pathophysiology of systemic osteoporosis in RA. The inflammatory cytokines TNF-a, IL-6, and IL-17 are responsible for localized osteoporosis in RA. In RA, IL-17 expression is unregulated, and the increased expression of Wnt10b and the Wnt-antagonist Dkk-1 was apparent. Thus, IL-17 plays an important role in bone pathophysiology in RA. Senescent T cells produce larger amounts of RNAKL in RA. Furthermore, anti-citrullinated protein antibodies (ACPAs) are associated with osteoporosis. In RA, glucocorticoid use, a low body mass index (BMI), low vitamin D, and disease activities are risk factors for osteoporosis.

Omata Y et al. [4] summarized the current evidence of the regulatory role of type 2 immunity in arthritis and bone metabolism, highlighting the role of type 2 cytokines and type 2 innate lymphoid cells (ILC2s). Type 2 immunity is exerted by T helper 2 (Th2) cells, Th 9 cells, eosinophils, mast cells, basophils, and ILC2s. IL-4 and IL-13, which are produced by type 2 immune cells, control osteoclast differentiation. The type 2 cytokines IL-4, IL-13,IL-5, IL-9, IL-25, and IL-33 exert regulatory effects on arthritis, reducing inflammation and inducing the resolution of arthritis. Type 2 cells also promote anti-osteoclastogenesis to stimulate osteoblasts to generate osteoprotegerin (OPG), which inhibits osteoclastogenesis. Type 2 immunity regulates arthritis and bone homeostasis via the Th2 cells ILC2s. The two immune cells have pleiotropic effects on immunity in the bone marrow and joints.

In RA, the synovial fluid is enriched by a subset of dendritic cells (DC) derived from monocytes (Mo-DCs), which promote deleterious Th17 responses. To date, in RA, four main environmental factors have been identified as impacting the differentiation/activation of Mo-DCs: (i) agonists of the aryl hydrocarbon receptor (AhR), (ii) extracellular acidosis, (iii) GM-CSF produced by synovial CD4+ T cells, and (iv) synoviocytes and synovial fluid. Coutant F [5] reviewed the characterization of environmental factors in joints and the fate of human Mo-DC. Mo-DCs are influenced by interactions with cytokines and deregulated synoviocytes. The citrullination process is also enhanced by osteoclastogenesis from Mo-CD. The identification of environmental mediators that control the differentiation of Mo-DC is also important. Activated Mo-DCs are potent inducers of Th17 cells that cause cartilage destruction and chronic inflammation and promote bone loss. Thus, identifying underlying molecular signaling pathways and environmental mediators that control the differentiation of Mo-DC can help develop new therapies for RA.

Tsuchiya H et al. [6] reviewed the recent progress in multifaceted analyses of synovial tissue in RA. The presence of autoantibodies (i.e., rheumatoid factor (RF) and anti-cyclic citrullinated peptide antibody (anti-CCP)) predicts a better response of anti-CD20 antibody and CTLA4 Ig. The body mass index (BMI) is associated with higher disease activity and disability in RA patients. Recently, ultrasound-guided needle biopsy has become widely accepted. The combination of histopathological findings and gene expression information has improved the prediction of biological therapy requirements. RA synovium was categorized into four major phenotypes according to the gene expression pattern: lymphoid, myeloid, low inflammatory, and fibroid. A higher myeloid score predicted a good clinical response to TNF-a inhibitors. The single-cell RNA sequencing of RA synovium led to the classification of synovial fibroblasts into at least three subpopulations. The distribution of synovial cell types shows biological differences among patients. Combining peripheral blood and synovial information could be feasible and lead to improved outcomes.

Yamasaki S et al. [7] reviewed the possible role of tRNA fragments in RA. They focused on the current state of noncoding RNA research in relation to RA, especially on tRNA fragments and their relevance to RA pathophysiology. Stress-induced small RNAs (tiRNAs) have a potential role in RA pathophysiology. tRNAs can exert antiapoptotic functions. As the RA phase shifts from preclinical to clinical, 5′ti RNAs with G-rich sequences have the ability to suppress global translation in synoviocytes and lymphocytes, which may reduce cytokine and protease production and suppress RA activity. As cytokine therapy has been used for RA, clarifying the association between cytokines and tiRNA activity may prove the importance of tiRNA in RA, and lead to new therapeutic strategies.

Extracellular DNA, called cell-free DNA (cfDNA), is elevated in peripheral blood and synovial fluid in the presence of RA and associated with disease activity. cfDNA is released into circulating blood following cell death, such as via apoptosis, necrosis, proptosis, ferroptosis, or extracellular trap-associated cell death, and mainly consists of double-stranded nuclear DNA and mtDNA. The profiling of cfDNA in patients with RA may help make a prognosis and predict treatment responses, and may be a biomarker of RA progression. Hashimonto T et al. [8] reviewed the sources cfDNA in patients with RA and the correlation of cfDNA with RA pathogenesis. It is suggested that neutrophil extracellular traps (NETs)/neutrophil etosis (NETosis) in the RA synovium produce cfDNA in RA fibroblast-like synoviocytes (FLSs). In addition, monocytes, macrophages, and FLSs are considered to release cfDNA in RA. Treatment with DMARDs and bDMARDs induced the apoptosis of RA FLSs; thus, cfDNA may be released after FLS apoptosis as a result of treatment. Research on cfDNA and its potential as a biomarker for disease progression and treatment responses is continuing to grow and is expected to contribute to personalized medicine.

Iwamoto N et al. [9] investigated the effect of methotrexate (MTX) on microRNA (miRNA) modulation in RA-FLS. miRNA, small endogenous RNAs that post-transcriptionally regulate the expression of genes, is a key regulator of biologic processes.

In this study, the authors performed miRNA array analysis to investigate differentially expressed miRNAs.

The microarray analysis identified 13 differentially expressed microRNAs in MTX-treated RA-FLS. To validate these microarray findings, they performed quantitative RT-PCR with additionally cultured fibroblasts, and among 13 miRNAs, miR-877-3p was consistently up-regulated in MTX-treated RA-FLS.

The authors demonstrated that (1) the expression of miR-877-3p was increased by the MTX treatment of RA-FLS, and (2) the up-regulation of miR-877-3p led to the decreased production of cytokines and chemokines and decreased cell migration. miR-877-3p down-regulated the expression of GM-CSF and CCL3 in RA-FLS. The administration of GM-CSF to RA patients led to disease flares, and CCL3 induced a variety of proinflammatory activities. According to the authors’ analysis, miR-877-3 has the potential to regulate RA-FLS through immune system and cell signaling regulations.

This brief report establishes a mechanism link between the anti-inflammatory, antimigratory effect of MTX on RA-FLS and the up-regulation of miR-877-3p. In addition, the up-regulation of miR-877-3p has the potential to provide a foundation for novel RA treatment via the modulation of RA-FLS.

Matsuda K et al. [10] reviewed the latest findings regarding the crosstalk between synovial fibroblasts and immune cells and the pivotal role of synovial fibroblasts in joint destruction in RA. The receptor activator of the NF-kB ligand (RANKL) is expressed in the inflamed synovium and induces bone destruction. State-of-the-art analytical technologies, such as single-cell RNA sequencing (scRNA-seq), have provided insight into the heterogeneity of synovial cells that produce the key factors. Synovial fibroblasts are the primary contributors to the joint destruction of RA and represent a promising therapeutic target. Cartilage destruction is a crucial aspect in the structural damage caused by RA, although clinical trials targeting the inhibition of MMPs, the cause of cartilage destruction, have failed because of musculoskeletal side effects. Combining single-cell transcriptomics with techniques such as mass cytometry may facilitate the identification of novel and detailed subsets of pathological synovial fibroblasts in RA and may lead to the development of personalized and novel therapies.

The citrullination of peptides that favor the production of antibodies against citrullinated proteins produced by bacteria has been reported. Currently, the gut microbiota is considered one of the key factors in the physiopathology of RA. Zaragoza-Garcia et al. [11] analyzed the relation between bacteria’s abundance, as well the bacterial ratios with different serological markers related to inflammation in RA. The serum levels of intestinal fatty-acid-binding protein 2 were higher in patients with secondary non-response to csDMARD. Thus, they suggest that the ratio of the gut microbiota’s bacteria and intestinal permeability seems to establish the precursor for therapeutic secondary non-response in RA.

It has been reported that among RA patients, organokines have been associated with increased inflammation and cartilage degradation due to augmented cytokines and metalloprotease production. Laurindo et al. [12] reviewed the role of adipokines, osteokines, myokines and hepatokines in RA progression. Changes in the pattern of organokine secretion directly and indirectly aggravate RA, and organokines implicate higher radiographic damage, immune dysregulation, and angiogenesis.

Organokines can act as RA potent regulators of cell proliferation, differentiation, and apoptosis, as well as immune cells’ chemotaxis to RA sites. The abovementioned review may contribute to developing new diagnostic and therapeutic methods of RA.

Oral microbiome changes take place at the initiation of RA. Arleevskaya M et al. [13] reported that anti-citrullinated peptide antibodies control periodontal bacteria such as oral porpyromonas and aggregatibacter species in patients with RA. In the RA-established cohort, *porpyromonas* sp. and *aggregatibacter* sp. reductions were associated with elevated ACPA levels. A decrease in oral porpyromonas sp was observed in ACPA-positive individuals, and this predominates in early-RA patients as compared to non-RA individuals, irrespective of their clinically suspected arthralgia score. Oral dysbiosis may help to determine individuals at high risk of developing RA.

RA and periodontitis are suggested to be closely linked based on microbial dysbiosis, although limited subgingival bacteria have been proven in the pathogenesis of RA. Chen YJ et al. [14] enrolled 30 RA patients and 25 controls divided into three groups: group AM (all of the matched participants), group PD (periodontally diseased) and group PH (periodontally healthy). Aminipila butyric and peptococus simile displayed a positive correlation with the level of anti-citrullinated protein antibodies (ACPAs) in the AM and PD groups. The authors elucidated the important role of aminipila butyric and peptococus simile as periodontal bacteria leading to RA through the induction of ACPA production.
